# Bronchial ethanol injection therapy for airway obstruction in lung cancer patients

**DOI:** 10.1002/rcr2.816

**Published:** 2021-07-29

**Authors:** Fukuko Okabe, Yukihiro Yano, Tomoki Kuge, Takeshi Uenami, Masaki Kanazu, Masahide Mori

**Affiliations:** ^1^ Department of Thoracic Oncology Osaka Toneyama Medical Center Osaka Japan

**Keywords:** airway obstruction, bronchial, ethanol injection therapy, low cost, lung cancer

## Abstract

Obstructive endobronchial tumours often cause decreased quality of life. Bronchial ethanol injection (BEI) therapy is considered an effective modality for airway dilatation or haemoptysis without specialist equipment. Here, we report experiences of two cases in which BEI therapy was effective for obstructive endobronchial tumours. In Case 1 with adenoid cystic carcinoma of the lung, BEI therapy and balloon dilatation were performed as treatment for the left main bronchus restenosis after metallic stent insertion. In Case 2 with squamous cell carcinoma of the lung, BEI therapy was performed after radiation therapy to the lesion that recurred in the entrance of the superior segment of the right lower lobe. Tumour progression was controlled with multiple BEI therapy. We consider BEI therapy useful because this procedure is easy to conduct, has low cost and can be done under particular conditions including post‐tracheobronchial stent placement and post‐radiation.

## Graphical abstract

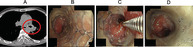

## INTRODUCTION

Obstructive endobronchial tumours often cause breathing impairment, haemoptysis and decreased quality of life if the airway patency cannot be maintained. It is necessary to take action for the improvement of subjective symptoms. There are options for treatment such as tracheobronchial stent placement, Nd:YAG laser therapy and radiation therapy for these conditions. However, these treatments need specialist equipment and require a certain level of skill. Furthermore, laser therapy is no longer effective once an endobronchial metallic stent has been inserted. Radiation therapy is also difficult to perform for lesions once radiation therapy has been done because of the dose limitation. Bronchial ethanol injection (BEI) therapy for obstructive endobronchial tumours is considered an effective modality for airway dilatation or haemoptysis without specialist equipment. This procedure can be done additionally after endobronchial stent insertion or radiation therapy.

Here, we report experiences of two cases in which BEI therapy was effective for obstructive endobronchial tumours. In Case 1 with adenoid cystic carcinoma of the lung, BEI therapy and balloon dilatation were performed as treatment for the left main bronchus restenosis after metallic stent insertion. In Case 2 with squamous cell carcinoma of the lung, BEI therapy was performed after radiation therapy to the lesion after recurrence in the entrance of the superior segment of the right lower lobe.

## CASE REPORT

### Case 1

**FIGURE 1:**
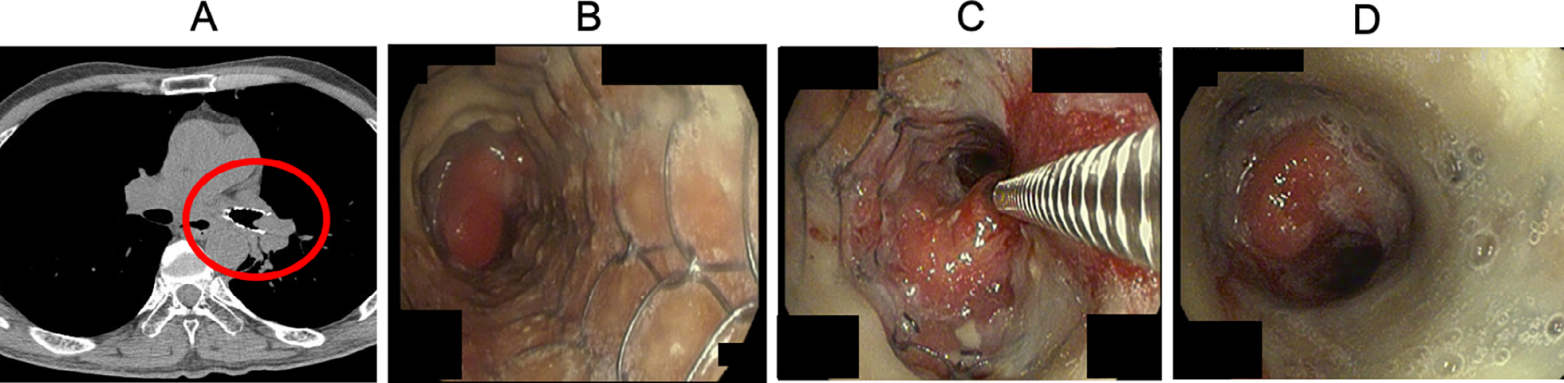
Chest computed tomography showing the narrowing airway at distal portion of the left main bronchus 2 years and 3 months after stent insertion (A). Bronchoscopic examination showing polypoid mass lesion at the distal end of the inserted stent (B). The procedure of bronchial ethanol injection (BEI) therapy with aspiration needle using absolute ethanol (C). Size reduction of polypoid mass lesion after BEI therapy (D)

A 64‐year‐old man presented our hospital with cough and dyspnoea. Chest computed tomography (CT) demonstrated a narrowed airway in the left main bronchus. Bronchoscopy showed stenosis of the left main bronchus. Histological examination revealed that it was adenoid cystic carcinoma of the lung. Chemoradiotherapy was performed as the first‐line treatment, then endoscopic metallic stent insertion was performed to prevent restenosis after chemoradiotherapy. The distal portion of the left main bronchus gradually stenosed at 2 years and 3 months after stent insertion. Chest CT showed narrowing airway at the distal portion of the left main bronchus (Figure 1A). Bronchoscopic examination revealed a polypoid mass lesion at the distal end of the inserted stent (Figure 1B). The bronchoscope could not pass through this lesion. There were no other recurrent lesions. Therefore, BEI therapy was performed to reduce tumour size. A dose of 0.1–0.8 ml of absolute ethanol (99.5% ethanol) was injected four times with aspiration needle over 8 months with the assistance of one‐time balloon dilatation (Figure 1C). As a result, polypoid mass size decreased and the bronchoscope could pass through (Figure 1D). However, the proximal left main bronchus became constricted again due to recurrence 1 year after the first BEI therapy. We decided to conduct chemotherapy as the next treatment and treated for approximately 2 years. After disease recurrence occurred, BEI therapy was performed in the same way as described previously with concurrent chemotherapy, with reduction in airway obstruction. No clinically meaningful treatment complication was experienced except for mild cough during the procedure. Four months after the last BEI therapy was performed, bronchoscopy showed tumour recurrence. We decided to conduct another chemotherapy as the next treatment. Tumour progression was controlled for approximately 1.5 years in total with multiple BEI therapy through the assistance of balloon dilatation and chemotherapy.

### Case 2

**FIGURE 2:**
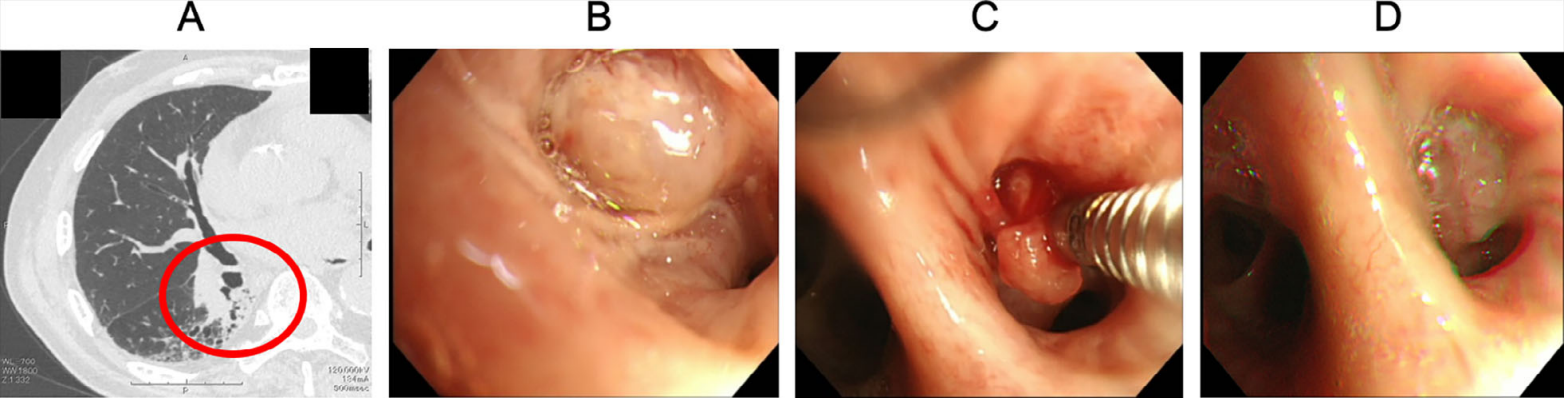
Chest computed tomography showing consolidation in the superior segment of right lung 5 years after external beam radiation therapy (A). Bronchoscopic examination showing polypoid mass lesion at the entrance of right B6 bronchi (B). The procedure of bronchial ethanol injection (BEI) therapy with aspiration needle using absolute ethanol (C). Size reduction of polypoid mass lesion 5 months after multiple BEI therapy (D)

A 73‐year‐old man had haemoptysis. Bronchoscopy showed the appearance of irregular tumour located around the distal portion of trachea to carina. Histological examination revealed squamous cell carcinoma of the lung. External beam radiation therapy was performed. The disease was stable without other treatment such as chemotherapy. Five years later, the patient represented with haemoptysis. Chest CT showed consolidation in the superior segment of the right lung (Figure 2A). Bronchoscopic examination revealed a polypoid mass lesion at the entrance of right B6 bronchi (Figure 2B), and consistent with recurrence. Radiation therapy was not performed again because the tumour recurred in the area of previous radiotherapy. There were no other recurrent lesions. Therefore, BEI therapy was performed for reduction of tumour size. A dose of 0.3–0.4 ml of absolute ethanol was injected with aspiration needle (Figure 2C). Five months after the procedure, the tumour size gradually reduced (Figure 2D). BEI therapy was performed on 10 occasions over a 2‐year period maintaining control of the tumour progression. No clinically meaningful treatment complication was experienced except for mild cough during the procedure. One month after the last BEI therapy performed, tumour recurrence was detected in the right lower lobe. We decided to conduct radiotherapy on the recurred lesion. Tumour progression was controlled for approximately 2 years with multiple BEI therapy.

## DISCUSSION

In the present cases, we described effective treatment with BEI therapy in maintaining stable condition for airway obstructions. The most important characteristic of the tumours in the presented cases was slow growth, which is one of the key factors in considering the indications of BEI therapy. In addition, we consider it important performing BEI therapy with the assistance of other modalities such as balloon dilatation and chemotherapy to achieve effectiveness in some cases.

BEI therapy has been performed for treating obstructive endobronchial tumours especially in Japan. In 1986, Fujisawa et al. reported 13 patients with malignant tracheobronchial lesions who received BEI therapy.^1^ This treatment was described as effective in polypoid tumour protruding into the tracheobronchial lumen but ineffective in case of compressed stenosis or obstruction. Change of bronchoscopic findings of the tumour surface from faintly white to necrotic within several days was also described. The same group reported the utility of BEI therapy not only for obstructive endobronchial tumour, but also for bleeding and tumour reduction.^2^ In 1991, Hamada et al. reported 10 patients with malignant tracheobronchial lesions who received BEI therapy.^3^ Four patients treated with BEI therapy had effective results; however, in three patients, BEI therapy was ineffective. Similar to the report from Fujisawa et al., they concluded that BEI therapy was effective not only for superficial or submucosally infiltrating lesions, but also for polypoid proliferative lesions. Other than these reports, there are several case reports from Japan that describe the utility of BEI therapy for airway obstruction in thoracic malignant disease.^4^ We found only one report from Egypt other than Japan that described BEI therapy for such diseases. ElBadrawy et al. reported 20 lung cancer patients with airway obstruction.^5^ Twenty patients were divided into two groups with ethanol injection therapy plus chemotherapy and chemotherapy alone. Significant improvement in symptoms and radiographic findings were observed in the patients treated with the ethanol injection therapy group compared to the chemotherapy alone group. They concluded that intra‐tumoural ethanol injection was effective and safe adjuvant to chemotherapy for tumour debulking. Regarding adverse events in BEI therapy, cough during the procedure was experienced in two cases. Previous reports described this complication and concluded it manageable.^1^, ^2^, ^3^, ^4^, ^5^ According to the procedure protocol, absolute ethanol was injected through aspiration needle of 1.8 mm diameter and 120 cm length. Injected volume was calculated according to the size and location of the tumour. These procedures were done every 2 weeks up to five times. Our procedure was performed according to the description in earlier reports from Japan. A dose of 0.1–0.8 ml of absolute ethanol was used according to the tumour size. Regarding treatment periods, our procedure was performed until the tumour size was controlled. The devices needed in this procedure are absolute ethanol and aspiration needle in addition to ordinary bronchoscopy. This means that the cost of the procedure is low compared to other procedures prevalent in clinical practice. The procedure itself is considered easy to perform for clinicians who have skills in performing ordinary bronchoscopy. BEI therapy was performed as our daily clinical practice and consent was obtained from patients as per ordinary bronchoscopy before the procedure. The treatment procedure and complications were explained before the treatment. According to the Japan Society for Respiratory Endoscopy in 2010, BEI therapy was performed 138 times at 13% (46 of 357 institutions) of Japanese institutions—1.5 times per year in one institution.^6^ This report shows BEI therapy is performed infrequently even in Japan, despite the fact that this procedure is considered easy to perform. Slow growth is one of the key factors in considering the indications of BEI therapy. In addition, we consider it important performing BEI therapy with the assistance of other modalities such as balloon dilatation and chemotherapy. BEI therapy has not been performed in patients with compressed stenosis or extrinsic obstruction.^1^, ^3^

In conclusion, we show BEI to be an effective therapy in our two cases to maintain control of large airway obstruction in lung cancer patients. We consider BEI therapy useful because this procedure is easy to perform, has low cost and can be done under particular conditions such as post‐tracheobronchial stent placement and post‐radiation therapy. Appropriate candidates for BEI therapy are not patients who have extrinsic stenosis or obstruction but those who have polypoid tumours with slow growth.

## CONFLICT OF INTEREST

None declared.

## ETHICS STATEMENT

Appropriate written informed consent was obtained for publication of this case report and accompanying images.

## AUTHOR CONTRIBUTIONS

All authors were responsible for the diagnosis, treatment and care of the patient. Fukuko Okabe and Yukihiro Yano wrote the original draft of the manuscript. Takeshi Uenami and Masaki Kanazu cared the patients and obtained the images through their clinical practice. Tomoki Kuge, Takeshi Uenami, Masaki Kanazu, Yukihiro Yano and Masahide Mori supervised the manuscript. All authors edited and finalized the final version.
